# A Web- and Mobile-Based Intervention for Women Treated for Breast Cancer to Manage Chronic Pain and Symptoms Related to Lymphedema: Results of a Randomized Clinical Trial

**DOI:** 10.2196/29485

**Published:** 2022-01-17

**Authors:** Mei Rosemary Fu, Deborah Axelrod, Amber A Guth, Joan Scagliola, Kavita Rampertaap, Nardin El-Shammaa, Jeanna M Qiu, Melissa L McTernan, Laura Frye, Christopher S Park, Gary Yu, Charles Tilley, Yao Wang

**Affiliations:** 1 School of Nursing-Camden Rutgers University Camden, NJ United States; 2 Department of Surgery School of Medicine New York University New York, NY United States; 3 NYU Laura and Isaac Perlmutter Cancer Center NYU Langone Health New York, NY United States; 4 Mount Sinai Hospital Mount Sinai Center for Nursing Research and Innovation New York, NY United States; 5 Rowan School of Osteopathic Medicine Stratford, NJ United States; 6 Harvard Medical School Harvard University Boston, MA United States; 7 Research Services Boston College Chestnut Hill, MA United States; 8 College of Global Public Health New York University New York, NY United States; 9 Rory Meyers College of Nursing New York University New York, NY United States; 10 Department of Electrical and Computer Engineering and Biomedical Engineering New York University Tandon School of Engineering New York, NY United States

**Keywords:** pain, lymphatic exercises, symptoms, lymphedema, breast cancer, health behavior, mHealth

## Abstract

**Background:**

The-Optimal-Lymph-Flow (TOLF) is a patient-centered, web- and mobile-based mHealth system that delivers safe, easy, and feasible digital therapy of lymphatic exercises and limb mobility exercises.

**Objective:**

The purpose of this randomized clinical trial (RCT) was to evaluate the effectiveness of the web- and mobile-based TOLF system for managing chronic pain and symptoms related to lymphedema. The primary outcome includes pain reduction, and the secondary outcomes focus on symptom relief, limb volume difference measured by infrared perometer, BMI, and quality of life (QOL) related to pain. We hypothesized that participants in the intervention group would have improved pain and symptom experiences, limb volume difference, BMI, and QOL.

**Methods:**

A parallel RCT with a control–experimental, pre- and posttest, and repeated-measures design were used. A total of 120 patients were recruited face-to-face at the point of care during clinical visits. Patients were randomized according to pain in a 1:1 ratio into either the arm precaution (AP) control group to improve limb mobility and arm protection or The-Optimal-Lymph flow (TOLF) intervention group to promote lymph flow and limb mobility. Trial outcomes were evaluated at baseline and at week 12 after the intervention. Descriptive statistics, Fisher exact tests, Wilcoxon rank-sum tests, t test, and generalized linear mixed effects models were performed for data analysis.

**Results:**

At the study endpoint of 12 weeks, significantly fewer patients in the TOLF intervention group compared with the AP control group reported chronic pain (45% [27/60] vs 70% [42/60]; odds ratio [OR] 0.39, 95% CI 0.17-0.90; *P*=.02). Patients who received the TOLF intervention were significantly more likely to achieve a complete reduction in pain (50% [23/46] vs 22% [11/51]; OR 3.56, 95% CI 1.39-9.76; *P*=.005) and soreness (43% [21/49] vs 22% [11/51]; OR 2.60, 95% CI 1.03-6.81; *P*=.03). Significantly lower median severity scores were found in the TOLF group for chronic pain (MedTOLF=0, IQR 0-1 vs MedAP=1, IQR 0-2; *P*=.02) and general bodily pain (MedTOLF=1, IQR=0-1.5 vs MedAP=1, IQR 1-3; *P*=.04). Compared with the AP control group, significantly fewer patients in the TOLF group reported arm/hand swelling (*P*=.04), heaviness (*P*=.03), redness (*P*=.03), and limited movement in shoulder (*P*=.02) and arm (*P*=.03). No significant differences between the TOLF and AP groups were found in complete reduction of aching (*P*=.12) and tenderness (*P*=.65), mean numbers of lymphedema symptom reported (*P*=.11), ≥5% limb volume differences (*P*=.48), and BMI (*P*=.12).

**Conclusions:**

The TOLF intervention had significant benefits for breast cancer survivors to manage chronic pain, soreness, general bodily pain, arm/hand swelling, heaviness, and impaired limb mobility. The intervention resulted in a 13% reduction (from 40% [24/60] to 27% [16/60]) in proportions of patients who took pain medications compared with the AP control group, which had a 5% increase (from 40% [24/60] to 45% [27/60]). A 12% reduction (from 27% [16/60] to 15% [9/60]) in proportions of patients with ≥5% limb volume differences was found in the TOLF intervention, while a 5% increase in the AP control group (from 40% [24/60] to 45% [27/60]) was found. In conclusion, the TOLF intervention can be a better choice for breast cancer survivors to reduce chronic pain and limb volume.

**Trial Registration:**

Clinicaltrials.gov NCT02462226; https://clinicaltrials.gov/ct2/show/NCT02462226

**International Registered Report Identifier (IRRID):**

RR2-10.2196/resprot.5104

## Introduction

### Background

Annually, more than 260,000 women are diagnosed with breast cancer, and currently there are more than 3.8 million breast cancer survivors in the United States [1]. Even years after cancer treatment, about 40% of women treated for breast cancer suffer daily from chronic pain and more than 50% of women report multiple distressing symptoms related to lymph fluid accumulation [2-5]. The abnormal accumulation of lymph fluid after breast cancer treatment is a result of obstruction or disruption of the lymphatic system associated with cancer treatment (eg, removal of lymph nodes or radiotherapy), influenced by patient personal factors (eg, obesity or higher BMI), and triggered by factors such as infections or trauma [6-8]. The accumulation of lymph fluid leads to chronic and various pain sensations (ie, pain/aching/soreness/tenderness) in the ipsilateral upper limb or body and other symptoms related to fluid accumulation defined as lymphedema symptoms [3,9].

While significantly more breast cancer survivors with a diagnosis of lymphedema experience pain (45.2%), tenderness (52.4%), aching (61.9%), or soreness (31%), a substantial amount of breast cancer survivors without a diagnosis of lymphedema also experience pain (40%), tenderness (47.3%), aching (30%), or soreness (32.7%) [9]. On average, breast cancer survivors without lymphedema report about 5 lymphedema symptoms while breast cancer survivors with lymphedema report 10 symptoms [9,10]. Despite current advances in cancer treatment, it is clear that many breast cancer survivors still face long-term postoperative challenges as a result of experiencing daily pain and lymphedema symptoms.

Pain and lymphedema symptoms are debilitating late complications that impact the breast cancer survivors’ quality of life (QOL) [2,3,5,11]. Persistent pain related to cancer treatment is considered a stressful complication because it is perceived as a constant reminder of cancer [2,12] and exerts tremendous limitations on breast cancer survivors’ daily living [2,5]. Pain and lymphedema symptoms can instigate fears and induce feelings of loss of control [2,3,5]. Specifically, the experience of pain, including tenderness, aching, or soreness, causes significant and unrelenting distress among breast cancer survivors [3]. Such distress is usually heightened when breast cancer survivors expect pain and symptoms related to lymphedema to disappear but instead stay as a “perpetual discomfort” [3] (p853). The negative impact of pain and lymphedema symptoms can be a source of considerable disability and psychological distress that negatively influences the patient’s daily living [2,3,11,12] and creates a tremendous burden on the health care system [13]. Nonetheless, in clinical practice pain and symptoms related to lymphedema are still underrecognized and undertreated.

While more research is needed to explore the exact etiology of persistent pain and lymphedema symptoms (eg, arm swelling, breast swelling, chest wall swelling, heaviness, firmness, tightness, stiffness, numbness, burning, stabbing, tingling, and limited limb movement), physiologically, the accumulation of lymph fluid in the affected area or limb may create undue pressure on nerves, producing feelings of pain, aching, tenderness, soreness, burning, tingling, stabbing, and numbness as well as inducing sensations of swelling, heaviness, tightness, and firmness [14,15]. Accumulated lymph fluid in the affected area or limb also leads to stiffness and limited limb movement of the arm, shoulder, fingers, and elbow [10,15]. Significant associations are found between pain (including aching and tenderness) and accumulation of lymph fluid in the ipsilateral upper limb [10,15]. Research has also shown that with the increased number of symptoms reported, breast cancer survivors’ limb volume increased [10,15]. Limb volume as detected by the infrared perometer has significantly elevated as breast cancer survivors’ reports of pain, tenderness, aching, swelling, heaviness, firmness, and tightness have increased [10]. For breast cancer survivors without a diagnosis of lymphedema, persistent pain and lymphedema symptoms are cardinal symptoms of early stage lymphedema because such symptoms often precede changes in limb size or girth or a lymphedema diagnosis [9]. Without a timely intervention, this early disease stage can progress into lymphedema that no surgical or medical interventions can cure [7,15].

Breast cancer survivors are known to have a compromised lymphatic system due to breast surgery, dissection of lymph nodes and vessels, and radiation, which leads to ineffective lymphatic drainage, thus accumulation of lymph fluid in the affected area or limb [10,15,16]. In addition to the risk factor of compromised lymphatic drainage from cancer treatment, higher BMI is also an established risk factor for the accumulation of lymph fluid [6-10]. Physiologically, a larger body mass creates a disproportion in lymph transport and capacity, resulting in excess extracellular fluid [6,17]. Women are 1.11 times more at risk for developing lymphedema with every 1 kg/m^2^ increase in their BMI [6-8,16]. Although the known risk factors for symptoms related to accumulation of lymph fluid directly from cancer treatment cannot be avoided (such as removal of lymph nodes, surgery, radiation, chemotherapy, and hormonal therapy), some risk factors (such as compromised lymphatic drainage and higher BMI) can be modified through education and self-care strategies [14,18,19].

Patient education focusing on self-care strategies holds great promise for reducing the risk of lymph fluid accumulation [14,18,19]. Research evidence demonstrates that even after controlling for confounding cancer treatment–related risk factors, patient education on self-care strategies remains an important predictor for patient-centered outcomes, including symptom experience and self-care behaviors [14,18,19]. Current patient education emphasizes precautionary lifestyle behaviors, such as avoidance of repetitive limb movement, lifting weighted objects, needle punctures, blood draw, and the use of compression garments for air travel in the affected limb [20,21]. To date, there is a paucity of high-quality evidence to support these precautionary practices that reduce the risk of lymphedema and relieve pain or symptoms related to lymph fluid accumulation [20,21]. Research is lacking to provide evidence to reduce pain and symptoms related to lymph fluid accumulation through self-care strategies targeting compromised lymphatic drainage and higher BMI.

Grounded in research-driven self-care behavioral strategies [14,19], The-Optimal-Lymph-Flow (TOLF) [22], a unique patient-centered web- and mobile-based educational and behavioral program, focuses on self-care strategies targeting compromised lymphatic system to promote lymph flow, limb mobility, and maintaining optimal BMI, that is, risk factors for pain and lymphedema symptoms. Patients learn self-care strategies through the web- and mobile-based program that can be downloaded on a computer, laptop, and any mobile phones and tablets. Its underlying premise is to empower, rather than inhibit, how breast cancer survivors live their lives by emphasizing “what to do,” rather than “what to avoid.” It features a safe, feasible, and easily integrated-into-daily-routine self-care strategies that include therapeutic lymphatic exercises (ie, muscle tightening–breathing, muscle tightening–pumping exercises, and large muscle exercises) to promote lymph flow and drainage, limb mobility exercises to promote shoulder and arm function, and general instructions to encourage nutrition-balanced (more vegetables and fruits), portion-appropriate diet (feeling 75% full for each meal), adequate hydration, and sleep to strive for maintaining optimal BMI. Patients can learn and follow all the exercises through avatar video simulations [14,19]. The efficacy of The Optimal Lymph-Flow has been demonstrated in our recently published study of 140 patients who received the face-to-face nurse-delivered program [19]. Findings of the study demonstrated that over 90% of patients improved their limb volume at the 12-month follow-up. This system has been used successfully for its usability testing. The preliminary usability and feasibility testing were completed with 30 breast cancer survivors who evaluated the easiness, difficulties, and feasibility of using the system on computer, iPhone, iPad, or other smartphones or tablets [23]. Findings of the usability and feasibility testing have demonstrated that patients love the web-based program, especially the videos using the avatar technology to demonstrate the complicated lymphatic system, and illustrate the physiological functions of each exercise and detailed step-by-step instructions for each exercise.

### Objectives and Hypotheses

The purpose of this randomized clinical trial (RCT) was to evaluate the efficacy of the web- and mobile-based TOLF system, a patient-centered educational and behavioral symptom management program focusing on promoting lymph flow, improving limb mobility, and optimizing BMI, for managing chronic pain and lymphedema symptoms.

The primary objective of this study was to determine the effectiveness of the web- and mobile-based TOLF system for managing chronic pain, aching, soreness, tenderness, and general bodily pain among breast cancer survivors. We hypothesized that more patients who received the TOLF intervention would report a complete reduction and reduced severity of pain, aching, soreness, tenderness, and general bodily pain compared with patients who received the arm precaution (AP) control at week 12 after the intervention.

The secondary objective of the study was to evaluate the effectiveness of the web- and mobile-based TOLF system for managing lymphedema symptoms, limb volume differences, BMI, and QOL related to pain. We hypothesized that patients who received the TOLF intervention would report fewer lymphedema symptoms, minimal limb volume differences, and better BMI and QOL compared with patients who received the AP control.

## Methods

### Ethical Approval

This study (IRB# i15-00221) was approved by the Institutional Review Board of New York University Langone Medical Center on June 8, 2015.

### Design

Chronic pain, including aching, soreness, and tenderness, is defined as persistent or intermittent pain in the ipsilateral upper limb or body at least 3 months after surgical treatment for breast cancer, that is, beyond the expected period of healing [24,25]. A 12-week, 2-arm, parallel RCT (ClinicalTrials.gov Identifier: NCT02462226) was designed to evaluate the effectiveness of the web- and mobile-based TOLF self-care strategies to promote lymph flow versus control AP for managing chronic pain and lymphedema symptoms. The data collectors were blinded to the group assignments. The protocol was in accordance with the CONSORT-EHEALTH checklist (Multimedia Appendix 1) [26].

### Setting

The study was conducted in a nursing research laboratory located in the breast cancer clinic of New York University Laura and Isaac Perlmutter Cancer Center, a National Cancer Institute–designated cancer center in New York City.

### Study Participants

Study participants included (1) patients who received surgical treatment for cancer at least 3 months prior to the study enrollment, because healing usually occurs within 3 months of surgical treatment for cancer [24,25]; (2) patients who reported *persistent or intermittent* pain (including aching, soreness, or tenderness); (3) patients who may or may not report any of lymphedema symptoms (ie, swelling, heaviness, tightness, firmness, numbness, tingling, stiffness, limb fatigue, limb weakness, and impaired limb mobility of shoulder, arm, elbow, wrist, and fingers); (4) patients who may or may not have a history of lymphedema or have been treated for lymphedema; (5) patients who had internet access to the web- and mobile-based program at home or were willing to access the program using the laptop provided by the researchers at the cancer center; (6) patients who had the ability to understand and the willingness to sign a written informed consent document.

Exclusion criteria were (1) patients who did not report any pain, including aching, soreness, or tenderness; (2) patients who had a known metastatic disease or other bulk disease in the thoracic or cervical regions; (3) patients who had lymphedema due to cancer recurrence; and (4) patients who had documented advanced cardiac or renal disease.

### Recruitment

**Figure 1:**
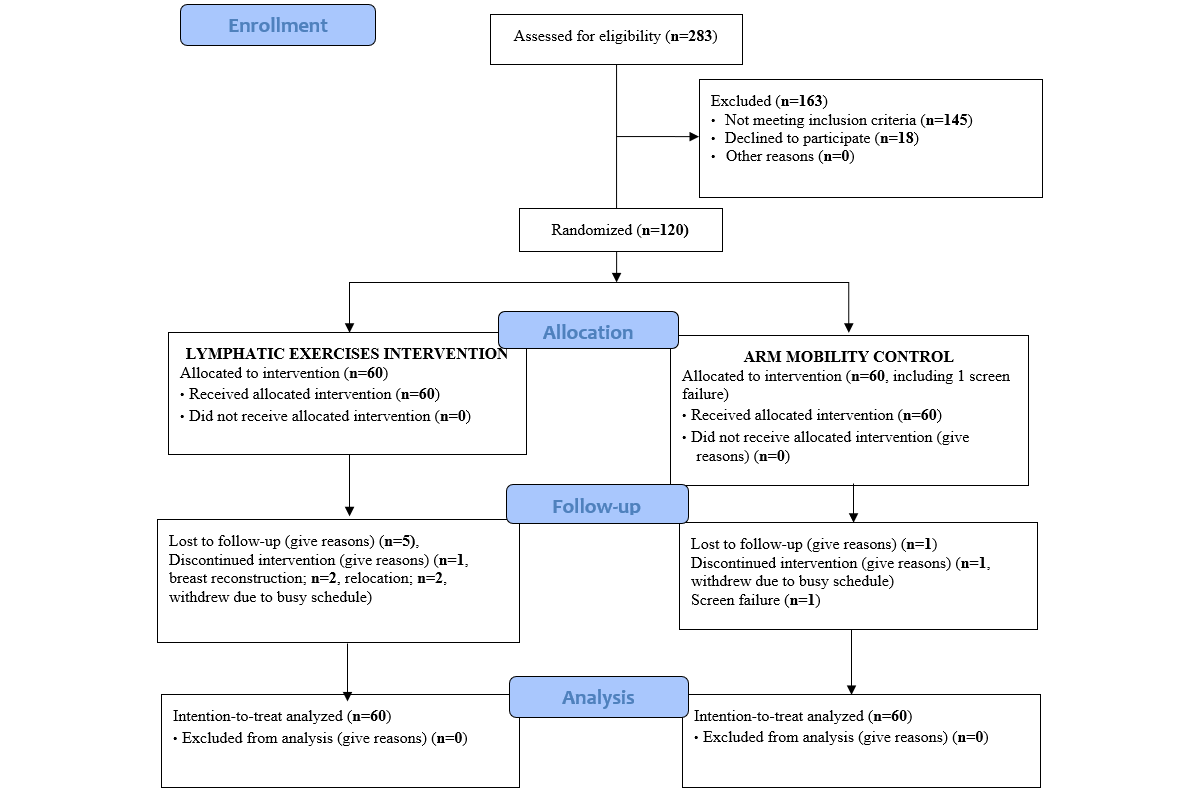
CONSORT-EHEALTH flowchart for recruitment.

From June 17, 2015, to December 1, 2016, we screened 283 patients for eligibility and enrolled and randomized 120 patients and followed the participants for 12 weeks after the intervention. Among the 283 patients screened, 163 were excluded for the following reasons: (1) not meeting inclusion criteria (n=145) and (2) declined to participate (n=18). Participants were recruited face to face at the point of care during clinical visits from the New York University Perlmutter Cancer Center. Figure 1 presents the CONSORT-EHEALTH diagram for recruitment and randomization.

To accomplish the recruitment of 120 participants, we used the successful procedures of recruiting and consenting participants used by the principal investigator and the team in the preliminary studies [3,9,14,17,19]. Successful strategies included the use of *invitation flyers* that described the study. This *invitation flyer* was posted on the bulletin boards or breast cancer support website at the cancer center, and was also available in the reception areas of the cancer center, examination rooms, and rooms holding support group meetings.

### Consent Process

After reading the *invitation flyer*, women who were interested in participating in the study called and scheduled a meeting with the research coordinator. During the meeting, the research coordinator confirmed the woman’s interest, determined if the woman was eligible for the study, and the research coordinator again explained the study in detail and provided enough time for the woman to ask questions. Protection of human participants was ensured by following the guidelines set forth by the Institutional Review Board. Each participant signed the written study consent.

### Randomization and Blinding

The randomization assignment was generated by our senior statistician (GY) using a computer-generated randomization procedure. Participants were randomized based on their report of pain/aching/soreness or tenderness to be allocated with a 1:1 ratio to either the TOLF intervention or the AP control group. The researchers who performed pre- and postintervention measurements were blinded throughout the study to the participants’ assigned arm. Participants did not know which intervention was the intervention of interest and which one was the comparator. Of the 120 patients enrolled, 60 were assigned to the TOLF intervention group and 60 to the AP control group (Figure 1).

### Study Intervention

#### Overview

The web- and mobile-based TOLF system [19,22,23] included information about lymphedema, diagnosis and measurement of lymphedema, lymphatic system, risk of lymphedema, self-care, daily therapeutic exercises, APs, and Ask Experts. Participants in the TOLF intervention group had access to the 8 avatar videos that provided step-by-step instructions for TOLF lymphatic exercises to promote lymph flow and optimize shoulder and limb mobility. The platform also has a section entitled *Arm Precautions*, representing current patient education that emphasizes precautionary lifestyle behaviors, such as avoidance of repetitive limb movement, lifting weighted objects, needle punctures, blood draw, and the use of compression garments for air travel in the affected limb [20,21]. Table 1 presents the strategies, rationales, and actions for the TOLF intervention.

**Table 1:** The-Optimal-Lymph-Flow Program: self-care strategies, rationales, and actions.

Strategies and exercises			Rationales			Actions
**Promoting lymph flow**						
	Muscle tightening deep breathing	The whole-body lymph fluid has to be drained through the lymphatic ducts above the heart. Muscle tightening–deep breathing stimulates lymphatic ducts and helps lymph fluid drain. Lymph fluid drains when muscles move. Muscle tightening–deep breathing creates the whole-body muscle movements that create muscle milking and pumping action and help to drain lymph fluid.		At least twice a day in the morning and at night before brushing teeth or as much as the patient wants throughout the day. Air travel: before take-off and after landing. Sedentary lifestyle: At least every 4 hours.		
	Muscle tightening–pumping	Muscle tightening–pumping exercises create arm muscle pumping. This helps lymph fluid flow and decreases the fluid build-up in the arms. Muscle tightening–pumping exercises build the arm muscle that helps lymph fluid flow and drain.		At least twice a day in the morning and at night before brushing teeth or as much as the patient wants throughout the day. Air travel: before take-off and after landing. Sedentary lifestyle: At least every 4 hours.		
**Improving limb mobility**						
	TOLF^a^ limb mobility exercises: shoulder rolls, clasp and spread, and reach to the sky. Arm precaution limb mobility exercises: shoulder rolls, clasp and spread, reach to the sky, wall climb, and sideway wall stretches.	Improved limb mobility after surgery facilitates local muscle movements that create muscle milking and pumping to promote local limb lymph fluid flow and drain. Shoulder exercises create arm muscle milking and pumping by moving the main anterior upper arm muscles (biceps brachii, brachialis, coracobrachialis), the posterior muscle of triceps brachii, and deltoid muscle (ie, the anterior deltoid, lateral deltoid, and posterior deltoid).			One week after surgery if there are no surgical drains or after the surgical drains are removed. At least twice a day until limb functions are returned to normal. Whenever limb mobility is limited throughout the recovery.	
**Keep a healthy weight**						
	Eat nutrition-balanced diet (ie, more vegetables and fruits as well as quality proteins). Maintain portion-appropriate diet (feeling 75% full for each meal).	Overweight or obesity is an important risk factor for lymph fluid accumulation. Having extra weight makes it difficult for lymph flow and drain. This can lead to extra lymph fluid build-up. There are numerous weight management programs available to assist with weight loss. Although there are a lot of weight reduction programs, each person may respond differently to each program. The core of the weight management is to eat a nutrition-balanced, portion-appropriate diet. It is also important to stay hydrated, exercise, and get adequate sleep.			Each meal daily It is important to talk to the nutritionist who can help to find a proper weight reduction program.	
	Stay hydrated	People may actually be thirsty, not hungry.			Drink 6-8 glasses of water daily; in the morning, before and during meals, and throughout the day. Avoid drinks with calories (eg, juices). Drink green tea to boost metabolism.	
	Large muscle exercises	Daily large muscle exercises (eg, walking, running, swimming, yoga) help to burn more calories. Daily large muscle exercises also promote lymph flow by creating muscle pumps.			At least 30 minutes 3 times a week or daily	
	Get enough sleep	Lack of sleep increases the production of the stress hormone cortisol, creates hunger, and leads to overeating. Getting just 1 more hour of sleep per night reduces belly fat accumulation.			At least 7-8 hours of sleep per night.	

^a^TOLF: The-Optimal-Lymph-Flow.

#### The-Optimal-Lymph-Flow Intervention Group (n=60)

Patients assigned to the TOLF intervention group were granted the access to the web- and mobile-based TOLF platform to learn about the program and therapeutic lymphatic exercises during the first in-person research visit. Patients had the access to the website contents of Lymphedema, Diagnosis of Lymphedema, Lymphatic System, Self-care, Therapeutic Lymphatic Exercises, and Ask Experts. Patients also had the access to the 8 avatar videos with step-by-step instructions to perform lymphatic exercises to promote lymph flow and optimize shoulder and limb mobility. In addition, the patients were introduced to an app, and had the choice to use either the web-based program or the app for practicing lymphatic exercises. However, patients in the TOLF intervention group did not have the access to the section *Arm Precautions* because the participants in the TOLF intervention group received comparable information regarding self-care as in the *Arm Precautions* section but with particular emphasis on “what to do,” rather than “what to avoid.”

#### The Arm Precaution Control Group (n=60)

Patients assigned to the control AP group had access to the web- and mobile-based Arm Precaution program to learn about the program and therapeutic limb mobility exercises to promote limb mobility during the first in-person research visit. The AP program also focused on precautionary lifestyle behaviors, such as avoidance of repetitive limb movement, lifting weighted objects, needle punctures, blood draw, and the use of compression garments for air travel in the affected limb [20,21]. Patients had access to the following contents of the website: Lymphedema, Diagnosis of Lymphedema, Risk of Lymphedema, Lymphatic System, 5 avatar videos for Therapeutic Limb Mobility Exercises to promote limb mobility, and Arm Precautions.

#### Duration of Intervention

It took 30-45 minutes for patients to learn all the sections of the program and about 10 minutes to learn the TOLF lymphatic exercises for the intervention group through 8 avatar videos. It took about 5 minutes to perform a set of TOLF daily exercises each time. Participants in the AP control group had access to 5 limb mobility exercise avatar videos and it took 3 minutes to perform a set of limb mobility exercises each time. We encouraged patients to perform the assigned exercises at least twice a day during the 12-week study period.

### Data Collection

#### Data Collection Procedures

##### Overview

Data were collected at baseline prior to the intervention, and at week 12 after the intervention. Data collection at each in-person time point took approximately 30 minutes. Within 1 week of enrollment for the clinical trial, patients had baseline assessment of pain and symptoms, limb volume difference, BMI, and QOL. The follow-up in-person assessment occurred at week 12 after the intervention.

##### Two In-Person Research Visits

Patients had 2 in-person research visits: (1) prior to the intervention: baseline assessment of pain and symptoms, limb volume difference, BMI, and QOL; and (b) week 12 postintervention assessment of pain and symptoms, limb volume difference, BMI, self-care behaviors, and QOL.

##### Two Online Assessments

Patients in the intervention and control groups received an email that provided a link to assess pain at weeks 4 and 8 after the intervention. Confidentiality of the patients was protected for the online assessment because patients used their study ID to access the online assessment.

#### Outcome Measures

##### Demographic and Medical Information

A structured tool was used to gather demographic and medical information and verified through reviewing participants’ medical records [14,17,19]. The demographic and medical information included age, types of surgeries, lymph nodes procedure, radiation, chemotherapy, time since surgery, lymphedema diagnosis, and pain medications prior to and at week 12 after the intervention.

##### Primary and Secondary Outcome Measures

Primary measure focused on pain that was assessed prior to and at week 12 after the intervention during in-person visits as well as at weeks 4 and 8 postintervention online assessment. Secondary measures included symptoms, limb volume difference (measured using an infrared perometer), BMI, and QOL. Limb volume difference (measured using an infrared perometer) and BMI were measured prior to and at week 12 after the intervention during in-person visits. QOL was assessed prior to and at week 12 after the intervention during in-person visits as well as at weeks 4 and 8 after the online assessment.

*Pain and Lymphedema Symptoms. The Lymphedema and Breast Cancer Symptom Experience Index(Part I*) is a valid and reliable self-report tool to assess chronic pain, including aching, soreness, tenderness, and additional symptoms related to lymph fluid accumulation or lymphedema (ie, arm swelling, breast swelling, chest wall swelling, heaviness, firmness, tightness, stiffness, burning, stabbing numbness, tenderness, stiffness, redness, blistering, and tingling [pins and needles]) [14,17,19]. A response frame of last 3 months was used for all participants to ensure the chronicity of symptom presence during the first in-person visit prior to the intervention. A response frame of 7 days was used during the second in-person visit at week 12 after the intervention. Each item was rated on a Likert scale from 0 (no presence of a given symptom) to 4 (greatest severity of a given symptom). For this study, a complete pain reduction was defined when a patient’s pain score was greater than 0 prior to the intervention and when the pain score was 0 at week 12 after the intervention.

##### Limb Volume Difference Measurement Using an Infrared Perometer

Perometry (350S; Juzo) was performed on each arm as it was held horizontally. The perometer maps a 3D graph of the affected and nonaffected extremities using numerous rectilinear light beams, and interfaces with a computer for data analysis and storage. A 3D limb image was generated and limb volume was calculated. This optoelectronic method has an SD of 8.9 mL (arm), <0.5% of limb volume with repeated measuring [17,19]. We used the following formula to calculate limb volume: limb volume difference percent = (affected limb volume – unaffected limb volume)/unaffected limb volume. An interlimb volume difference of >10% is a widely accepted diagnostic criterion for breast cancer–related lymphedema [10], yet it is known that a 5% difference in interlimb volume causes symptoms [24,25] and impairments in activities of daily living [27]. Therefore, we used the interlimb volume difference >5% as the threshold for minimal limb volume differences in this study.

##### General Bodily Pain and Quality of Life Related to Pain

The 6-item Pain Impact Questionnaire (PIQ-6), a reliable and valid 6-question health survey, was used to measure the impact of pain on an individual’s functional health and well-being. The PIQ-6 measures the severity of general bodily pain and its impact on work and leisure activities, as well as on emotional well-being within a variety of diseases and general populations. High PIQ-6 *t*-scores indicate greater pain impact/worse health [28].

##### Height, Body Weight, and BMI

Height was measured to the nearest 0.1 cm with a portable stadiometer (Scale-Tronix 5002 Stand on Scale; Scale-Tronix Company) without shoes [29]. An electrical device (InBody 520, Biospace Co., Ltd) was used to measure the participants’ body weight, and BMI was calculated using the formula: weight (kg)/height (m^2^) [29].

##### Practice of Self-care Behaviors

The Risk Reduction Behavior Checklist, a structured self-report checklist, was used to quantitatively assess patients’ self-report of adherence to the assigned interventions at the study endpoint of 12 weeks after the intervention [17,19]. The checklist included a list of self-care behaviors that promote lymph flow (eg, muscle tightening–deep breathing, muscle tightening–pumping, limb mobility exercises).

### Statistical Analysis

#### Primary Endpoint

The primary endpoint for the study was a complete pain reduction or reduced pain severity reported by the participants at week 12 after the intervention.

#### Sample Size and Power Calculations

The target sample size was 120 participants to account for a potential attrition of 20%, which has been observed in previous studies on breast cancer survivors [10]. This allowed to yield an adequate analytic sample size even with 20% attrition based on a 2-sample 2-sided *t* test with α=.05 and power of 90% to detect a group difference of 0.7 SDs in pain severity reported by the participants at week 12 after the intervention. The projected sample size of 96 would also provide sufficient statistical power for mixed regression models and for linear mixed models of continuous outcomes (eg, QOL).

#### Data Analysis

Data downloading and entry were performed independently by 2 researchers who were not involved in data collection and had no conflicts of interest. Moreover, the data analysis was independently assessed by 2 experienced statisticians (MM and LF) who were not involved in the data collection. Data were analyzed using R version 3.6.2 (R Foundation for Statistical Computing). Descriptive statistics were performed for baseline demographic and clinical characteristics using parametric (eg, independent samples *t* test) and nonparametric tests (eg, chi-square test) as appropriate. All the tests were 2 tailed. Descriptive statistics were also performed to summarize the distributions for primary and secondary outcome variables.

As planned [30], Fisher exact tests were used to test the primary hypothesis that more patients who received the TOLF intervention would report a complete reduction of chronic pain, aching, soreness, tenderness, and general bodily pain compared with patients who received the AP intervention at week 12 after the intervention. Wilcoxon rank sum tests were performed to test the hypothesis that patients who received the TOLF intervention would report less severe chronic pain, aching, soreness, tenderness, and general bodily pain at week 12 after the intervention compared with patients who received the AP control intervention. The proportion of patients reporting complete pain reduction was compared between the TOLF intervention and AP control groups prior to the intervention and at week 12 after the intervention using Fisher exact tests.

As planned [30], additional mixed effects models were conducted to test for between-group differences in change of pain over the study period. Generalized linear mixed effects models (cumulative logit mixed models) were used to analyze ordinal outcomes (eg, ratings pain, aching, soreness, tenderness, general bodily pain) and generalized linear mixed models (binomial mixed effects models with a logit link) were used to analyze binary outcomes (presence of pain, aching, soreness, tenderness, general bodily pain). These models incorporated fixed effects for time, treatment group, and a time × group interaction term, as well as a random intercept to account for repeated within-person observations. The models were estimated using maximum likelihood with adaptive Gaussian quadrature approximation methods.

To test the secondary hypothesis that patients who received the TOLF intervention would report fewer lymphedema symptoms, minimal limb volume differences, and better BMI and QOL compared with patients who received the AP control intervention, independent sample *t* tests for numeric continuous variables and chi-square or Fisher exact tests for nominal variables were used to assess the changes between the group differences in secondary outcomes between the TOLF intervention and AP control groups at week 12 after the intervention. We supplemented each of these comparisons with between-group tests prior to the intervention for reference.

#### Method of Handling Missing Data and Nonadherence to Protocol

There was no case of nonadherence to study protocol. No participants had a missing data >20%. Data were missing from the 6 patients due to attrition. Other participants have intermittent missing data throughout the study due to nonresponse. All missing data were not systematic but missing at random. The primary objective of this RCT required nonparametric tests, precluding the use of Rubin’s rules for multiple imputation and intent-to-treat analysis [31]. These results were all based on complete cases, and inferences represent effects of treatment on the treated. In addition, linear mixed effects models with maximum likelihood estimation were used to address between-group differences in pain, aching, soreness, and tenderness during the intervention. These analyses were in accordance with intent-to-treat principles [31].

## Results

### Participant Characteristics at Baseline

Among the 120 enrolled patients, 114 participants completed the study, including 1 case of screen failure. This patient in the control group was deemed ineligible but completed the study because she was diagnosed with other cancer before the end of the study (0.8% [1/114] screen failure). At week 12 after the intervention, 5 participants in the intervention group and 1 participant in the control group did not complete the study (5% [6/120] attrition rate). There were no statistical differences in demographic and treatment characteristics between patients that completed the study and the 6 patients who did not. No statistical differences were also found between participants in the TOLF intervention and AP control groups in terms of demographic and treatment characteristics except that participants in the TOLF intervention group had higher weight compared with those in the AP control group at baseline prior to the intervention. As a result, the randomization scheme based on the presence of pain, aching, soreness, or tenderness created 2 relatively similar patient profiles (Table 2).

As shown in Table 2, at baseline, the participants were women with a mean age of 56.7 years (SD 10.6; range 54.7-58.6). More than 70% (88/120, 73.3%) had a bachelor or graduate/professional degree, 50.8% (61/120) were married, and 65.8% (79/120) were employed. Of the 120 patients, 70% (84/120) had lumpectomy while 30% (36/120) had mastectomy, 59.2% (71/120) had chemotherapy, and 77.5% (93/120) had radiation therapy. While 32.5% (39/120) of the patients underwent axillary lymph nodes dissection, 59.2% (71/120) had sentinel lymph nodes biopsy alone. The mean lymph nodes removed was 7.3 (SD 7.7; range 5.9-9.0). Only 15.8% (19/120) of the participants had been diagnosed with and treated for lymphedema. There was a 13% reduction (from 40% [24/60] to 27% [16/60]) in proportions of patients who took pain medications in the TOLF intervention compared with a 5% increase (from 40% [24/60] to 45% [27/60]) in the control group at week 12 after the intervention.

**Table 2:** Demographic and clinical characteristics of participants at baseline (N=120).

Characteristics			Total (N=120), mean (SD), range		Arm precaution (n=60)		The-Optimal-Lymph-Flow (n=60)		Statistics (*df*)		*P* value^a^
Age (years), mean (SD), range			56.7 (10.6), 54.7-58.6		56.8 (11.0), 53.9-59.6		56.6 (10.3), 53.9-59.2		*t*_116.69_=–0.11		.91
Body weight (lb), mean (SD), range			163.58 (38.2), 156.6-170.5		156.2 (39.0), 146.1-166.2		171.1 (36.2), 161.7-180.5		*t*_116.59_=2.17		.03
Number of lymph nodes removed, mean (SD), range			7.3 (7.7), 5.9-9.0		7.4 (8.7), 5.1-9.7		7.2 (6.5), 5.5-9.0		*t*_103.46_=–0.11		.91
Time since breast cancer diagnosis (years) to study enrollment, mean (SD), range			2.8 (1.2), 2.6-3.0		2.7 (1.2), 2.4-3.0		2.9 (1.2), 2.5-3.2		*t*_114.65_=0.62		.54
**Level of education, n (%)**									Fisher exact test (5)		.79
	High school or below	26 (21.7)		12 (20.0)		14 (23.3)					
	Associate’s degree	6 (5.0)		3 (5.0)		3 (5.0)					
	Bachelor’s degree	47 (39.2)		23 (38.3)		24 (40.0)					
	Master’s degree	29 (24.2)		17 (28.3)		12 (20.0)					
	Doctoral degree	7 (5.8)		2 (3.3)		5 (8.3)					
	Professional degree	5 (4.2)		3 (5.0)		2 (3.3)					
**Marital status, n (%)**									Fisher exact test (4)		.16
	Married	61 (50.8)		26 (43.3)		35 (58.3)					
	Divorced/separated	16 (13.3)		11 (18.3)		5 (8.3)					
	Widowed	7 (5.8)		3 (5.0)		4 (6.7)					
	Partnered	9 (7.5)		3 (5.0)		6 (10.0)					
	Single or never partnered	27 (22.5)		17 (28.3)		10 (16.7)					
**Ethnicity, n (%)**									Fisher exact test (4)		.98
	Asian	10 (8.3)		5 (8.3)		5 (8.3)					
	African American or Black	22 (18.3)		12 (20.0)		10 (16.7)					
	White	72 (60.0)		35 (58.3)		37 (61.7)					
	Hispanic/Latino	11 (9.2)		5 (8.3)		6 (10.0)					
	More than 1 race	5 (4.2)		3 (5.0)		2 (3.3)					
**Employment status, n (%)**									*χ*_1_^2^=0.07		.80
	Unemployed	41 (34.2)		19 (32)		22 (36.7)					
	Employed	79 (65.8)		41 (68)		38 (63.3)					
**Mastectomy**									*χ*_1_^2^=0.07		.84
	Yes	36 (30.0)		19 (31.7)		17 (28.3)					
	No	84 (70.0)		41 (68.3)		43 (71.7)					
**Lumpectomy**											>.99
	Yes	60 (50.0)		30 (50.0)		30 (50.0)					
	No	60 (50.0)		30 (50.0)		30 (50.0)					
**Being diagnosed with and treated for lymphedema, n (%)**									Fisher exact test (1)		.32
	Yes	19 (15.8)		12 (20.0)		7 (11.7)					
	No	98 (81.7)		47 (78.3)		51 (85.0)					
**Radiotherapy, n (%)**									*χ*_1_^2^=1.13		.29
	Yes	93 (77.5)		44 (73.3)		50 (83.3)					
	No	27 (22.5)		16 (26.7)		10 (16.7)					
**Chemotherapy, n (%)**									*χ*_1_^2^=0.412		.52
	Yes	71 (59.2)		33 (55.0)		38 (63.3)					
	No	49 (40.8)		27 (45.0)		22 (36.7)					
**Axillary lymph nodes dissection, n (%)**									*χ*_1_^2^<0.001		>.99
	Yes	39 (32.5)		20 (33.3)		19 (31.7)					
	No	27 (22.5)		14 (23.3)		13 (21.7)					
**Sentinel lymph nodes biopsy alone, n (%)**									Fisher exact test (1)		.71
	Yes	71 (59.2)		40 (66.7)		41 (68.3)					
	No	49 (40.8)		20 (33.3)		19 (31.7)					
**Taking pain medications at baseline, n (%)**									*χ*_1_^2^<0.001		>.99
	Yes	48 (40.0)		24 (40.0)		24 (40.0)					
	No	72 (60.0)		36 (60.0)		36 (60.0)					
**Taking pain medications at week 12 after the intervention, n (%)**									*χ*_1_^2^=0.344		.56
	Yes	43 (35.8)		27 (45.0)		16 (26.7)					
	No	76 (63.3)		33 (55.0)		44 (73.3)					

^a^*P* values were derived from independent samples *t* tests for numeric outcomes. For categorical outcomes, *P* values correspond to chi-square tests of independence unless any cell sizes are <10, in which case a Fisher exact test was performed.

### Complete Reduction of Pain, Aching, Soreness, Tenderness, and General Bodily Pain

As shown in Table 3, at baseline prior to the intervention, there were no significant differences between the TOLF intervention and AP control groups in terms of proportions of patients who reported chronic pain (*P*>.99), aching (*P*=.42), soreness (*P*=.12), tenderness (*P*=.28), and general bodily pain (*P*>.37). At the study endpoint of week 12, significantly fewer patients in the TOLF intervention group compared with the AP control group reported chronic pain (45% [27/60] vs 70% [42/60]; odds ratio [OR] 0.39, 95% CI 0.17-0.90; *P*=.02). No significant differences were found between the TOLF and AP groups in terms of proportion of patients who reported aching (*P*=.05), soreness (*P*=.12), or tenderness (*P*=.25) as well as general bodily pain (*P*=.28).

As presented in Table 4, Fisher exact tests demonstrated that patients who received the TOLF intervention were more likely to experience a complete reduction in chronic pain (50% [23/46] vs 22% [11/51]); OR 3.56, 95% CI 1.39-9.76; *P*=.005) and soreness (43% [21/49] vs 22% [11/51]; OR 2.60, 95% CI 1.03-6.81; *P*=.03) compared with patients who received the AP control at week 12 after the intervention. There were no significant differences in complete reduction of aching (*P*=.12), tenderness (*P*=.65), and general bodily pain (*P*=.16) between the TOLF and AP groups.

**Table 3:** Proportion of patients that reported chronic pain, soreness, aching, or tenderness, and general bodily pain at baseline prior to the intervention and at study endpoint of week 12 after the intervention.

Outcome variables			Arm precaution (n=60)				The-Optimal-Lymph-Flow (n=60)					Fisher exact test of independence^a^		
			No pain, n (%)		Pain, n (%)		No pain, n (%)		Pain, n (%)		Odds ratio (95% CI)			*P* value
**Baseline prior to the intervention**														
	Chronic pain	8 (13)		52 (87)		9 (15)		51 (85)		0.87 (0.27-2.77)			>.99	
	Soreness	5 (8)		55 (92)		6 (10)		54 (90)		0.82 (0.19-3.44)			>.99	
	Aching	6 (10)		53 (88)		10 (17)		49 (82)		0.56 (0.15-1.84)			.42	
	Tenderness	8 (13)		52 (87)		12 (20)		47 (78)		0.61 (0.20-1.77)			.34	
	General bodily pain	3 (5)		57 (95)		1 (2)		59 (98)		1.04^b^ (0.97-1.11)			.37	
**Week 12 after the intervention**														
	Chronic pain	17 (28)		42 (70)		28 (47)		27 (45)		0.39 (0.17-0.90)			.02^c^	
	Soreness	17 (28)		42 (70)		24 (40)		31 (52)		0.53 (0.22-1.22)			.12	
	Aching	17 (28)		42 (70)		26 (43)		28 (47)		0.44 (0.19-1.01)			.05	
	Tenderness	19 (32)		40 (67)		24 (40)		31 (52)		0.62 (0.27-1.41)			.25	
	General bodily pain	11 (18)		48 (80)		15 (25)		40 (67)		0.89^b^ (0.73-1.09)			.28	

^a^Degrees of freedom (*df*)=1 for all the tests.

^b^Because general bodily pain is a very likely outcome prior to the intervention, we report risk ratio rather than odds ratio for this outcome at both time points. The 95% CI corresponds to the risk ratio, and *P* values are obtained using Monte Carlo simulation.

^c^Statistical significance.

**Table 4:** Proportions of patients with complete pain reduction between the intervention and control groups (“Yes”= complete pain reduction) using Fisher exact tests.

Complete pain reduction	Arm precaution		The-Optimal-Lymph-Flow			Test of group differences		
	No, n (%)^a^	Yes, n (%)	No, n (%)	Yes, n (%)	OR^b^ (95% CI)		*P* value	NNT^c^
Chronic pain	40 (78)	11 (22)	23 (50)	23 (50)	3.56 (1.39-9.76)		.005^d^	3.6
Tenderness	38 (75)	13 (25)	29 (69)	13 (31)	1.31 (0.48-3.56)		.65	16.7
Soreness	42 (78)	11 (22)	28 (57)	21 (43)	2.60 (1.03-6.81)		.03^e^	4.8
Aching	40 (77)	12 (23)	26 (60)	17 (40)	2.16 (0.82-5.86)		.12	5.9
General bodily pain	48 (86)	8 (14)	40 (74)	14 (26)	2.09 (0.73-6.36)		.16	8.3

^a^Percentage is based on numbers of patients who reported chronic pain, tenderness, soreness, aching, and general bodily pain at baseline. That is, denominator for each symptom is different. For example, for chronic pain for the arm precaution group N is 51. There were 51 patients in the arm precaution group who reported nonzero chronic pain at visit 1.

^b^OR: odds ratio, a measure of effect size. Recommended interpretation: 1.5=small, 2=medium, 3=large. Degrees of freedom (*df*)=1 for all the tests.

^c^NNT: number needed to treat, that is, the number of patients who would need to participate in the TOLF intervention (instead of the AP control) for 1 additional patient to experience a complete pain reduction.

^d^Significant at the *P*<.01 level.

^e^Significant at the *P*<.05 level.

### Severity of Chronic Pain, Aching, Soreness, Tenderness, and General Bodily Pain

At baseline, there were no significant differences in terms of median severity of chronic pain (*P*=.08), aching (*P*=.05), soreness (*P*=.07), tenderness (*P*=.13), or general bodily pain (*P*=.56) between the TOLF intervention and AP control groups (Table 5). At week 12 after the intervention, the TOLF group had significantly lower median severity scores for chronic pain (Med_TOLF_=0, IQR=0-1 vs Med_AP_=1, IQR 0-2; *P*=.02) and general bodily pain (Med_TOLF_=1, IQR 0-1.5 vs Med_AP_=1, IQR=1-3; *P*=.04).

**Table 5:** Severity of chronic pain, soreness, aching, or tenderness, and general bodily pain as well as quality of life (PIQ-6) at baseline prior to the intervention and study endpoint of week 12 after the intervention.

Outcome variables			Arm precaution (n=60), median (IQR)		The-Optimal-Lymph-Flow (n=60), median (IQR)		Independent samples test for between-group differences				
							Wilcoxon *r*^a^ (95% CI)		W-score		*P* value
**Baseline prior to the intervention**											
	Chronic pain	2 (1-3)		1 (1-2)		0.161 (–0.029 to 0.334)		2125		.08	
	Soreness	2 (1-3)		2 (1-2)		0.165 (–0.008 to 0.346)		2133		.07	
	Aching	2 (1-3)		2 (1-2)		0.177 (–0.008 to 0.346)		2089		.05	
	Tenderness	2 (1-3)		2 (1-3)		0.139 (–0.035 to 0.328)		2048		.13	
	General bodily pain	2 (1.75-3)		2 (2-3)		0.054 (–0.138 to 0.233)		1908.5		.56	
	Quality of life^b^ by PIQ-6^c^	56.1 (9.3)		54.1 (7.5)		0.234 (–0.125 to 0.601)		1.30 (112.9)		.20	
**Week 12 after the intervention**											
	Chronic pain	1 (0-2)		0 (0-1)		0.206 (0.030 to 0.378)		2001		.02^d^	
	Soreness	1 (0-2)		1 (0-2)		0.117 (–0.071 to 0.292)		1837		.20	
	Aching	1 (0-2)		1 (0-1.75)		0.160 (–0.032 to 0.335)		181.5		.08	
	Tenderness	1 (0-2)		1 (0-2)		0.055 (–0.121 to 0.237)		1723.5		.55	
	General bodily pain	1 (1-3)		1 (0-1.5)		0.188 (0.016 to 0.355)		1968		.04^d^	
	Quality of life^b^ by PIQ-6	50.7 (8.1)		48.4 (7.9)		0.290 (–0.088 to 0.669)		1.53 (108.5)		.13	

^a^Wilcoxon *r*: Measure of effect size. Recommended interpretation: 0.1=small, 0.3=medium, 0.5=large.

^b^For quality of life, data in columns 2-6 are presented as mean (SD), mean (SD), Cohen *d* (95% CI), t-score (*df*), and *P* value. Cohen *d* is a measure of effect size. Recommended interpretation: 0.2=small, 0.5=medium, 0.8=large.

^c^PIQ-6: 6-item Pain Impact Questionnaire.

^d^Significant at the *P*<.05 level.

### Changes of Pain, Aching, Soreness, Tenderness, and General Bodily Pain

Cumulative link mixed effects models were used to predict the ordinal pain outcomes (pain, soreness, aching, tenderness) across the 4 measurement time points and to determine group differences in the changes during the study time. As shown in Multimedia Appendix 2, there was a significant decrease in severity of chronic pain, aching, soreness, and tenderness for both the TOLF intervention and AP control groups across the 4 time points (baseline, week 4, 8, and 12 after the intervention). There was no significant time × group interaction effect for chronic pain (*P*=.14), aching (*P*=.23), soreness (*P*=.22), and tenderness (*P*=.18).

Binomial mixed effects models were used to assess group differences in the prevalence of chronic pain, aching, soreness, and tenderness across the study time points. Model results (Multimedia Appendix 3) indicate that patients were less likely to experience chronic pain, tenderness, soreness, and aching throughout the course of the study. This effect was consistent for both the TOLF intervention and AP control groups. There were no group differences.

A cumulative link mixed effects model was also used to predict severity of general bodily pain across the 4 time points and to determine whether the 2 groups differed in how pain scores vary across the 4 study time points. As Multimedia Appendix 4 shows, there was a significant decrease in general bodily pain across the 4 time points. This effect was consistent for both the TOLF intervention and AP control groups. There was no significant time × group interaction (*P*=.22). Results of the binomial mixed effects model to assess group differences in the prevalence of general bodily pain across the study period are shown in Multimedia Appendix 4. Patients were less likely to experience general bodily pain throughout the course of the study. This effect was similar between the TOLF intervention and AP control groups.

### Quality of Life Related to Pain

At baseline prior to the intervention, there was no significant difference (*t*_112.9_=1.30, *P*=.20) in mean QOL by PIQ-6 scores between the TOLF intervention (54.1 [SD 7.5]) and AP control (56.1 [SD 9.3]). At the study endpoint of week 12, there was no significant difference in mean QOL by PIQ-6 scores (*t*_108.5_=1.53, *P*=.13) between the TOLF intervention (48.4 [SD 7.9]) and the AP control (50.7 [SD 8.1]). As more improvement in the PIQ-6 scores was found in the TOLF intervention group at week 12 after the intervention, we conducted a subsequent linear mixed effects model predicting PIQ-6 scores across the 4 study time points and confirmed that PIQ-6 scores were significantly improved during the study (*B*=–1.73, 95% CI –2.33 to –1.13, *P*<.001) in the TOLF and AP groups, and that changes in PIQ-6 were not statistically different between the TOLF intervention and AP control groups (*b*=0.07, 95% CI *–*0.80 to 0.93; *P*=.88; Multimedia Appendix 5).

### Lymphedema Symptoms, Limb Volume Differences, and BMI

Table 6 presents the occurrence of the 23 lymphedema symptoms at baseline prior to and after the intervention. Compared with the AP control group, at week 12 after the intervention, significantly fewer patients in the TOLF intervention group reported arm/hand swelling (*P*=.04), heaviness (*P*=.03), redness (*P*=.03), and limited movement in shoulder (*P*=.02) and arm (*P*=.03). As shown in Table 7, there were no significant differences at week 12 after the intervention between the TOLF intervention and AP control groups in terms of mean numbers of lymphedema symptom reported, ≥5% limb volume differences, and BMI. There was a 12% reduction (from 27% [16/60] to 15% [9/60]) in the proportion of patients with ≥5% limb volume differences from baseline to postintervention in the TOLF group, while there was a 5% increase (from 40% [24/60] to 45% [27/60]) in the proportion of patients with ≥5% limb volume differences from baseline to postintervention in the AP group.

**Table 6:** Lymphedema symptom occurrence at baseline and after the intervention.

Lymphedema symptoms			Arm precaution (n=60), n (%)		The-Optimal-Lymph-Flow (n=60), n (%)		*P* value^a^
**Arm/hand swelling**							
	Baseline	34 (57)		30 (50)		.46	
	Week 12 after the intervention	28 (47)		17 (28)		.04^b^	
**Breast swelling**							
	Baseline	23 (38)		30 (50)		.57	
	Week 12 after the intervention	14 (23)		13 (22)		.83	
**Chest wall swelling**							
	Baseline	7 (12)		10 (17)		.36	
	Week 12 after the intervention	8 (13)		8 (13)		>.99	
**Firmness in the affected limb**							
	Baseline	19 (32)		16 (27)		.57	
	Week 12 after the intervention	21 (35)		16 (27)		.55	
**Tightness in the affected limb**							
	Baseline	29 (48)		30 (50)		.49	
	Week 12 after the intervention	24 (40)		22 (37)		.76	
**Heaviness in the affected limb**							
	Baseline	26 (43)		20 (33)		.40	
	Week 12 after the intervention	26 (43)		15 (25)		.03^b^	
**Toughness of thickness of skin in the affected limb**							
	Baseline	14 (23)		9 (15)		.35	
	Week 12 after the intervention	10 (17)		8 (13)		.60	
**Stiffness in the affected limb**							
	Baseline	25 (42)		22 (37)		.61	
	Week 12 after the intervention	29 (48)		18 (30)		.10	
**Hotness/increased temperature in the affected limb**							
	Baseline	13 (22)		9 (15)		.47	
	Week 12 after the intervention	14 (23)		7 (12)		.13	
**Redness in the affected limb**							
	Baseline	7 (12)		4 (7)		.38	
	Week 12 after the intervention	8 (13)		1 (2)		.03^b^	
**Blistering in the affected limb**							
	Baseline	3 (5)		1 (2)		.62	
	Week 12 after the intervention	0 (0)		0 (0)		N/A^c^	
**Numbness in the affected limb**							
	Baseline	20 (33)		31 (52)		.01	
	Week 12 after the intervention	18 (30)		19 (32)		.96	
**Burning in the affected limb**							
	Baseline	4 (7)		12 (20)		.03	
	Week 12 after the intervention	3 (5)		9 (15)		.10	
**Stabbing in the affected limb**							
	Baseline	13 (22)		10 (17)		.65	
	Week 12 after the intervention	13 (22)		8 (13)		.19	
**Tingling (pins and needles) in the affected limb**							
	Baseline	25 (42)		32 (53)		.40	
	Week 12 after the intervention	20 (33)		23 (38)		.74	
**Fatigue in the affected limb**							
	Baseline	23 (38)		31 (52)		.29	
	Week 12 after the intervention	21 (35)		29 (48)		.15	
**Weakness in the affected limb**							
	Baseline	35 (58)		22 (37)		.05	
	Week 12 after the intervention	34 (57)		21 (35)		.02^b^	
**Seroma (pocket or fluid developed)**							
	Baseline	10 (17)		8 (13)		.76	
	Week 12 after the intervention	9 (15)		3 (5)		.08	
**Limited movement in shoulder**							
	Baseline	23 (38)		22 (37)		.84	
	Week 12 after the intervention	28 (47)		15 (25)		.02^b^	
**Limited movement in elbow**							
	Baseline	9 (15)		6 (10)		.48	
	Week 12 after the intervention	11 (18)		5 (8)		.15	
**Limited movement in wrist**							
	Baseline	15 (25)		11 (18)		.53	
	Week 12 after the intervention	15 (25)		7 (12)		.07	
**Limited movement in fingers**							
	Baseline	21 (35)		14 (23)		.27	
	Week 12 after the intervention	17 (28)		9 (15)		.08	
**Limited movement in arm**							
	Baseline	26 (43)		27 (45)		.46	
	Week 12 after the intervention	27 (45)		15 (25)		.03^b^	

^a^Chi-square tests of independence unless any cell sizes are <10, in which case a Fisher exact test was performed.

^b^Significant at *P*<.05.

^c^N/A: not applicable.

**Table 7:** Outcomes of lymphedema symptoms, limb volume differences, and BMI at baseline and after the intervention.^a^

Secondary outcome variables			The-Optimal-Lymph-Flow (n=60)	Arm precaution (n=60)	Statistics	*P* value^b^
**Number of lymphedema symptoms**						
	Baseline		9.2 (5.4), 7.8-10.6	10.6 (4.9), 9.3-11.8	*t*_116.96_=–1.48	.14
	Week 12 after the intervention		6.1 (5.1), 4.6-7.4	7.6 (5.2), 6.2-8.9	*t*_111.61_=–1.62	.11
**Mean BMI**						
	Baseline prior to the intervention		29.2 (6.0), 27.6-30.8	27.1 (6.4), 25.4-28.8	*t*_115.86_=1.86	.07
	Week 12 after the intervention		29.3 (6.3), 27.6-31.1	27.4 (6.5), 5.7-29.1	*t*_107.49_=1.58	.12
**≥5% Limb volume differences^c^**					*χ*_1_^2^=0.230	
	Baseline					.63
		Yes	16 (27)	13 (22)		
		No	44 (73)	47 (78)		
	Week 12 after the intervention^d^					.48
		Yes	9 (15)	16 (27)		
		No	51 (85)	44 (73)		

^a^Data are presented as mean (SD), range or n (%).

^b^*P* values are derived from independent samples *t* tests for numeric outcomes. For categorical outcomes, *P* values correspond to chi-square tests of independence unless any cell sizes are <10, in which case a Fisher exact test was performed.

^c^Limb volume difference percent = (affected limb volume – unaffected limb volume)/unaffected limb volume.

^d^Fisher exact test (*df*=1) was applied.

### Self-report of Adherence

Participants reported no adverse events of performing the TOLF lymphatic exercises and limb mobility exercises. In terms of self-reported adherence to the assigned interventions, 87% (52/60) of participants reported performing the TOLF lymphatic exercises twice a day as prescribed, while 83% (50/60) of participants reported performing limb mobility exercises twice a day as prescribed.

## Discussion

### Preliminary Findings

The therapeutic lymphatic and limb mobility exercise intervention is an essential component of the TOLF self-care pain management program [19,23]. The efficacy of the TOLF intervention relies on skill-based training in teaching patients to correctly perform the set of therapeutic exercises. Prior research identified that ambiguous and inadequate information was a barrier to initiate and maintain exercise for breast cancer survivors [32]. Clear information about how exercise should be done and how often it should be done is essential for patients to initiate and adhere to the prescribed therapeutic exercise regimen [33,34]. Extending prior research findings [23,35], this RCT provided additional evidence that the web- and mobile-based TOLF system is feasible and efficacious in training patients to perform lymphatic and limb mobility exercises via avatar videos with step-by-step instructions.

A recent a single-arm feasibility clinical trial with a pre- and posttest design to assess the effects of the TOLF therapeutic lymphatic exercise intervention demonstrated that a single session of a Kinect-enhanced TOLF intervention immediately reduces pain, swelling, and lymphedema symptoms in breast cancer survivors [35]. This current RCT was the first to evaluate the effectiveness of the web- and mobile-based TOLF system for managing chronic pain and lymphedema symptoms by comparing 2 parallel interventions. Results of this RCT demonstrated that the TOLF intervention to promote lymph flow led to more complete pain reductions and pain severity reductions at week 12 after the intervention compared with the AP control to improve limb mobility. The TOLF intervention achieved a large effect for complete reduction in pain (OR 3.56, 95% CI 1.39-9.76; *P*=.005) and a medium effect for complete reduction in soreness (OR 2.60, 95% CI 1.03-6.81; *P*=.03).

Current pain management relies heavily on pharmacological agents, such as opioids and nonsteroidal anti-inflammatory drugs [30,31], which were also the major pain medications that our participants took. It is important to note that a 13% reduction (from 40% [24/60] to 27% [16/60]) was observed in proportions of patients who took pain medications at week 12 after the intervention in the TOLF intervention, while a 5% increase in the AP control (from 40% [24/60] to 45% [27/60]) was noted. This result is promising due to concerns of poor efficacy, abuse, and adverse effects of opioids and nonsteroidal anti-inflammatory drugs [36,37]. Results of this RCT extend findings of prior single-arm trials [19,35] and suggest that the TOLF intervention is superior to the AP control in pain management.

Managing pain and lymphedema symptoms is critical to reduce the risk of lymphedema. Breast cancer survivors who report pain on the affected ipsilateral upper limb or body are nearly twice as likely to develop lymphedema [9]. For breast cancer survivors without a diagnosis of lymphedema, the experience of pain and lymphedema symptoms is a cardinal sign of subclinical lymphedema [38,39]. In this RCT, only 15.8% (19/120) of participants were diagnosed with or treated for lymphedema, yet all the participants without a diagnosis of or treated for lymphedema reported chronic pain and lymphedema symptoms at baseline prior to the intervention. Symptoms of arm/hand swelling, heaviness, redness, and limited movement in shoulder are hallmarks of fluid accumulation [28]. In this RCT, significantly fewer patients in the TOLF intervention group reported arm/hand swelling, heaviness, redness, and limited movement in shoulder and arm at the end of the trial. Extending findings of prior single-arm clinical trials [35,39], this RCT suggests that the TOLF intervention may be more effective than AP to effectively manage pain and lymphedema symptoms.

The TOLF lymphatic exercises were designed to decrease lymph fluid levels. In a previous study [19], 97% of the 134 patients who received the face-to-face TOLF intervention maintained or decreased their preoperative limb volumes assessed using an infrared perometer at 12 months after surgery. It is important to note that a 12% reduction (from 27% [16/60] to 15% [9/60]) in proportions of patients with ≥5% limb volume differences was observed in the TOLF group, whereas a 5% increase in the AP group (from 40% [24/60] to 45% [27/60]) was observed. This finding suggests that the TOLF intervention may be more effective in reducing limb volume than the AP control. In a recent study, significant reductions were found in lymph fluid levels assessed using bioimpedance immediately after a single training session of a Kinect-enhanced TOLF intervention [35]. More importantly, greater reductions in lymph fluid levels were found in patients with abnormal lymph fluid levels. The use of bioimpedance for assessing lymph fluid level may be a more sensitive measure than limb volume measurement using a perometer and should be applied in future studies.

### Strengths and Limitations

The strengths of the RCT are a safe novel digital intervention targeting the lymphatic system for chronic pain, 5% (6/120) attrition, rigorous study design with a larger sample size over 100 patients, and the consecutively identified participants with chronic pain. The use of technologically driven digital therapy not only enhanced the fidelity and transparency of the intervention delivery but also the reproducibility of the intervention, which may enhance the generalizability and dissemination of the intervention. The technologically driven delivery model enhanced the patients’ ability to learn to perform the assigned exercise therapy given that they were able to review the assigned exercise therapy on their own schedule and pace virtually anytime and anywhere. Another strength was the daily 5-minute routine of TOLF lymphatic exercises, which was easy for patients to establish in their own routine.

There were fewer limitations of this RCT. In our study, 87% [52/60] patients reported performing the TOLF lymphatic exercises twice a day as prescribed and 83% [50/60] reported performing AP limb mobility exercises. Lack of real-time monitoring limited the study’s ability to explore dose sensitivity for pain. Future study may use wearable devices to monitor patients’ adherence. Accumulation of lymph fluid in the affected area or limb leads to chronic inflammation resulting in pain for breast cancer survivors [28,29]. Pain following breast cancer treatment is significantly associated with the inflammatory cytokine gene *IL13* and lymphatic gene *VEGFC* [24,25]. Future research should investigate the genetic impact on the TOLF intervention as well as the efficacy of TOLF on the genetic expression of biomarkers.

### Conclusions

The results of this RCT showed significant benefits of the TOLF intervention for chronic pain, soreness, general bodily pain, and specific lymphedema symptoms (ie, arm/hand swelling, heaviness, limited movement in shoulder and arm) among breast cancer survivors in comparison with the AP control. The TOLF intervention resulted in a 13% reduction (from 40% [24/60] to 27% [16/60]) in proportions of patients who took pain medications compared with the AP control, which had a 5% increase (from 40% [24/60] to 45% [27/60]). In addition, a 12% reduction (from 27% [16/60] to 15% [9/60]) in proportions of patients with ≥5% limb volume differences was observed in the TOLF group and a 5% increase in proportions of patients in the AP group (from 40% [24/60] to 45% [27/60]). These findings suggest that the TOLF intervention should be a better choice for pain management and limb volume reduction in comparison to the AP control. The TOLF intervention is safe, efficacious, and affordable as a replacement or complement therapy for chronic pain management for millions of breast cancer survivors. The low-cost, detailed description of interventions, and technologically driven delivery model of the TOLF make it relatively easy to implement TOLF in clinical practice or at home.

## Appendix

Multimedia Appendix 1 CONSORT-EHEALTH checklist.

Multimedia Appendix 2 Results of the cumulative link mixed effects models for ordinal pain outcomes. Time is centered at baseline prior to intervention=0.

Multimedia Appendix 3 Results of the binomial mixed effects models with logit link for binary pain outcomes (0=no, 1=yes). Time is centered at baseline prior to intervention=0.

Multimedia Appendix 4 Results for the cumulative link mixed effects model for ordinal general bodily pain (left) and the binomial mixed effects model for prevalence (0=no, 1=yes) of general bodily pain (right). Time is centered at baseline prior to intervention=0.

Multimedia Appendix 5 Results from a linear mixed effects model predicting PIQ-6 (Quality of Life) scores. Time is centered at baseline prior to the intervention=0.

## Abbreviations

AP: arm precaution
mHealth: mobile health
OR: odds ratio
PIQ: Pain Impact Questionnaire
QOL: quality of life
RCT: randomized clinical trial
TOLF: The-Optimal-Lymph-Flow
