# Supplementary material for: A Web- and Mobile-Based Intervention for Women Treated for Breast Cancer to Manage Chronic Pain and Symptoms Related to Lymphedema: Results of a Randomized Clinical Trial
Source: JMIR Cancer. 2022 Jan 17;8(1):e29485. doi: 10.2196/29485 (PMC8893593; doi:10.2196/29485)
Supplement: Multimedia Appendix 2 [file cancer_v8i1e29485_app2.docx]

**Multimedia Appendix 2.** Results of the cumulative link mixed effects models for ordinal pain outcomes. Time is centered at baseline prior to intervention=0.

|  | **Chronic Pain** | | | **Tenderness** | | | **Soreness** | | | **Aching** | | |
| --- | --- | --- | --- | --- | --- | --- | --- | --- | --- | --- | --- | --- |
| *Predictors** | *Odds Ratios* | *CI* | *p* | *Odds Ratios* | *CI* | *p* | *Odds Ratios* | *CI* | *p* | *Odds Ratios* | *CI* | *p* |
| 0\|1 | 0.03 | 0.01 – 0.07 | <0.001 | 0.03 | 0.01 – 0.07 | <0.001 | 0.03 | 0.01 – 0.06 | <0.001 | 0.02 | 0.01 – 0.06 | <0.001 |
| 1\|2 | 0.36 | 0.16 – 0.79 | 0.011 | 0.32 | 0.14 – 0.73 | 0.007 | 0.29 | 0.14 – 0.61 | 0.001 | 0.29 | 0.13 – 0.68 | 0.004 |
| 2\|3 | 2.24 | 1.01 – 4.97 | 0.048 | 1.96 | 0.86 – 4.49 | 0.111 | 1.74 | 0.84 – 3.60 | 0.135 | 1.96 | 0.85 – 4.50 | 0.115 |
| 3\|4 | 20.87 | 8.00 – 54.47 | <0.001 | 26.52 | 9.74 – 72.22 | <0.001 | 19.84 | 7.67 – 51.30 | <0.001 | 39.88 | 13.11 – 121.33 | <0.001 |
| Time | 0.56 | 0.44 – 0.70 | <0.001 | 0.50 | 0.39 – 0.64 | <0.001 | 0.52 | 0.41 – 0.66 | <0.001 | 0.48 | 0.38 – 0.62 | <0.001 |
| Group (AP=0, TOLF=1) | 0.44 | 0.15 – 1.31 | 0.142 | 0.46 | 0.15 – 1.44 | 0.183 | 0.54 | 0.20 – 1.45 | 0.220 | 0.50 | 0.16 – 1.56 | 0.231 |
| Time*Group | 0.96 | 0.69 – 1.34 | 0.799 | 1.23 | 0.88 – 1.72 | 0.232 | 1.03 | 0.75 – 1.43 | 0.844 | 1.02 | 0.72 – 1.43 | 0.926 |
| **Random Effects** | | | | | | | | | | | | |
| σ^2^ | 3.29 | | | 3.29 | | | 3.29 | | | 3.29 | | |
| τ_00_ | 3.14 | | | 3.89 | | | 1.90 | | | 3.95 | | |
| ICC | 0.49 | | | 0.54 | | | 0.37 | | | 0.55 | | |
| N | 120 | | | 120 | | | 120 | | | 120 | | |
| Observations | 412 | | | 410 | | | 413 | | | 409 | | |
| Marginal R^2^ / Conditional R^2^ | 0.100 / 0.540 | | | 0.067 / 0.572 | | | 0.108 / 0.434 | | | 0.101 / 0.592 | | |

AP: arm precaution control group

TOLF: The-Optimal-Lymph-Flow intervention group

*Note: The odds-ratio for the threshold ‘0|1’ for chronic pain outcome suggests that the odds of having a chronic pain score of 0 is 0.03 times the odds of having a score of 1,2,3, or 4 at baseline prior to intervention for the AP control group. Similarly, the odds-ratio for the threshold ‘2|3’ for Chronic Pain outcome suggests that the odds of having chronic pain score of 0,1, or 2 is 2.24 times the odds of having a score of 3, or 4 at baseline prior to intervention for the AP control group.
