# Supplementary material for: A Web- and Mobile-Based Intervention for Women Treated for Breast Cancer to Manage Chronic Pain and Symptoms Related to Lymphedema: Results of a Randomized Clinical Trial
Source: JMIR Cancer. 2022 Jan 17;8(1):e29485. doi: 10.2196/29485 (PMC8893593; doi:10.2196/29485)
Supplement: Multimedia Appendix 3 [file cancer_v8i1e29485_app3.docx]

**Multimedia Appendix 3.** Results of the binomial mixed effects models with logit link for binary pain outcomes (0=no, 1=yes). Time is centered at baseline prior to intervention=0.

|  | **Chronic Pain** | | | **Tenderness** | | | **Soreness** | | | **Aching** | | |
| --- | --- | --- | --- | --- | --- | --- | --- | --- | --- | --- | --- | --- |
| *Predictors* | *Odds Ratios* | *CI* | *p* | *Odds Ratios* | *CI* | *p* | *Odds Ratios* | *CI* | *p* | *Odds Ratios* | *CI* | *p* |
| (Intercept) | 16.46 | 5.89 – 45.97 | **<0.001** | 11.15 | 4.31 – 28.87 | **<0.001** | 19.87 | 7.10 – 55.61 | **<0.001** | 35.70 | 8.75 – 145.66 | **<0.001** |
| Time | 0.62 | 0.44 – 0.89 | **0.010** | 0.63 | 0.45 – 0.89 | **0.009** | 0.57 | 0.39 – 0.82 | **0.003** | 0.52 | 0.35 – 0.79 | **0.002** |
| Group (AP=0, TOLF=1) | 0.63 | 0.19 – 2.11 | 0.455 | 0.86 | 0.25 – 2.92 | 0.811 | 1.02 | 0.29 – 3.60 | 0.976 | 0.61 | 0.13 – 2.93 | 0.539 |
| Time*Group | 0.69 | 0.43 – 1.13 | 0.143 | 0.99 | 0.61 – 1.60 | 0.960 | 0.73 | 0.43 – 1.21 | 0.223 | 0.74 | 0.42 – 1.30 | 0.288 |
| **Random Effects** | | | | | | | | | | | | |
| σ^2^ | 3.29 | | | 3.29 | | | 3.29 | | | 3.29 | | |
| τ_00_ | 3.07 | | | 3.65 | | | 2.73 | | | 6.90 | | |
| ICC | 0.48 | | | 0.53 | | | 0.45 | | | 0.68 | | |
| N | 120 | | | 120 | | | 120 | | | 120 | | |
| Observations | 412 | | | 410 | | | 413 | | | 409 | | |
| Marginal R^2^ / Conditional R^2^ | 0.120 / 0.545 | | | 0.042 / 0.546 | | | 0.117 / 0.517 | | | 0.099 / 0.709 | | |

AP: arm precaution control group

TOLF: The-Optimal-Lymph-Flow intervention group
