# Supplementary material for: A Web- and Mobile-Based Intervention for Women Treated for Breast Cancer to Manage Chronic Pain and Symptoms Related to Lymphedema: Results of a Randomized Clinical Trial
Source: JMIR Cancer. 2022 Jan 17;8(1):e29485. doi: 10.2196/29485 (PMC8893593; doi:10.2196/29485)
Supplement: Multimedia Appendix 4 [file cancer_v8i1e29485_app4.docx]

**Multimedia Appendix 4.** Results for the cumulative link mixed effects model for ordinal general bodily pain (left) and the binomial mixed effects model for prevalence (0=no, 1=yes) of general bodily pain (right). Time is centered at baseline prior to intervention=0.

| Predictors | General bodily pain severity | | | General bodily pain prevalence | | |
| --- | --- | --- | --- | --- | --- | --- |
|  | Odds ratios | CI | *P* value | Odds ratios | CI | *P* value |
|  |  |  |  |  |  |  |
| 0\|1 | 0.01 | 0.00-0.02 | **<.001** |  |  |  |
| 1\|2 | 0.15 | 0.07-0.33 | **<.001** |  |  |  |
| 2\|3 | 0.76 | 0.35-1.64 | .49 |  |  |  |
| 3\|4 | 5.74 | 2.54-12.95 | **<.001** |  |  |  |
| 4\|5 | 176.54 | 34.67-898.86 | **<.001** |  |  |  |
| Time | 0.53 | 0.42-0.67 | **<.001** | 0.52 | 0.31-0.88 | **.01** |
| Group (AP^a^=0, TOLF^b^=1) | 0.88 | 0.31-2.53 | .82 | 1.73 | 0.24-12.45 | .59 |
| Time × Group | 0.86 | 0.62-1.19 | .37 | 0.64 | 0.31-1.31 | .23 |
| (Intercept) |  |  |  | 127.54 | 13.54-1201.33 | **<.001** |
| **Random effects** | | | | | | |
| σ^2^ | 3.29 | | | 3.29 | | |
| τ_00_ | 2.89 | | | 6.81 | | |
| ICC | 0.47 | | | 0.67 | | |
| N | 120 | | | 120 | | |
| Observations | 413 | | | 413 | | |
| Marginal R^2^/conditional R^2^ | 0.109/0.526 | | | 0.098/0.706 | | |

Note: Bold values indicate significance.

^a^AP: arm precaution control group.

^b^TOLF: The-Optimal-Lymph-Flow intervention group.
