# Supplementary material for: A Web- and Mobile-Based Intervention for Women Treated for Breast Cancer to Manage Chronic Pain and Symptoms Related to Lymphedema: Results of a Randomized Clinical Trial
Source: JMIR Cancer. 2022 Jan 17;8(1):e29485. doi: 10.2196/29485 (PMC8893593; doi:10.2196/29485)
Supplement: Multimedia Appendix 5 [file cancer_v8i1e29485_app5.docx]

**Multimedia Appendix 5.** Results from a linear mixed effects model predicting PIQ-6 (Quality of Life) scores. Time is centered at baseline prior to the intervention=0.

| Predictors | PIQ-6 QoL score | | |
| --- | --- | --- | --- |
|  | Estimates | CI | *P* value |
|  |  |  |  |
| (Intercept) | 55.24 | 53.29 to 57.19 | **<0.001** |
| Time | –1.73 | –2.33 to –1.13 | **<0.001** |
| Group (AP^a^=0, TOLF^b^=1) | –1.66 | –4.42 to 1.11 | 0.24 |
| Time × Group | 0.07 | –0.80 to 0.93 | 0.88 |
| **Random effects** | | | |
| σ^2^ | 26.89 | | |
| τ_00_ _studyid_ | 39.36 | | |
| ICC | 0.59 | | |
| N _studyid_ | 120 | | |
| Observations | 412 | | |
| Marginal R^2^/conditional R^2^ | 0.063/0.620 | | |

Note: Bold values indicate significance.

^a^AP: arm precaution control group.

^b^TOLF: The-Optimal-Lymph-Flow intervention group.
